# Le syndrome d’Usher: à propos d’une observation

**DOI:** 10.11604/pamj.2017.27.217.5460

**Published:** 2017-07-21

**Authors:** Chama Daoudi, Noureddine boutimzine, Samia El Haouzi, Omar Lezrek, Samira Tachfouti, Mounir Lezrek, Mina Laghmari, Rajae Daoudi

**Affiliations:** 1 Université Mohammed V Souissi, Service d’Ophtalmologie A de l’Hôpital des Spécialités, Centre Hospitalier Universitaire, Rabat, Maroc

**Keywords:** cécité, surdité, rétinopathie pigmentaire, Blindness, deafness, pigmentary retinopathy

## Abstract

Le syndrome d'Usher est une maladie génétique comportant une double atteinte sensorielle (auditive et visuelle) appelée surdicécité. Nous rapportons l'observation d'un patient de 50 ans, issue d'un mariage consanguin présentant une surdité congénitale avec une fonction vestibulaire normale et une rétinopathie pigmentaire responsable d'une baisse bilatérale de l'acuité visuelle apparue vers l'âge de 16 ans. Cette association compose le type 2 du syndrome d'Usher, affection rare de transmission autosomique récessive. La chirurgie de la cataracte a permis une amélioration de l'acuité visuelle chez ce patient.

## Abstract (en)

Usher syndrome is a genetic disease resulting in double sensory deprivation (auditory and visual) called deafblindness. We report the case of a 50-year old patient, born to consanguineous parents, presenting with congenital deafness associated with normal vestibular function and pigmentary retinopathy responsible for decreased bilateral visual acuity occurred at the age of 16 years. This association composes Usher syndrome type 2, a rare autosomal recessive disorder. Cataract surgery allowed visual acuity improvement in this patient.

## Introduction

De nombreux syndromes associent une atteinte simultanée des systèmes visuel et auditif, le syndrome d'Usher en représente la cause génétique la plus fréquente de l'association cécité-surdité. Sa prévalence est de l'ordre de 1/25 000 et représente 3 à 6% des patients sourds et 18% de ceux atteints de rétinite pigmentaire.

## Patient et observation

**Figure 1:**
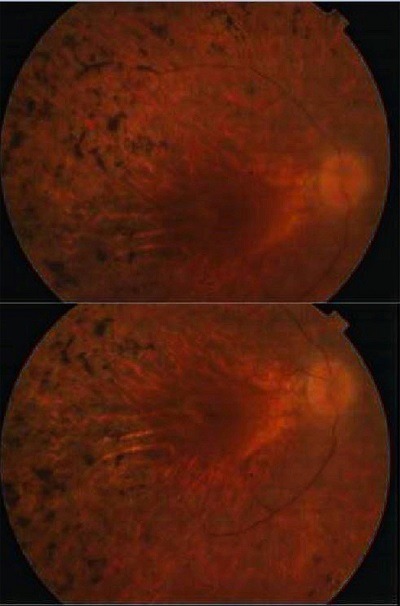
Photographie du fond d’œil de rétinopathie pigmentaire au cours d’un syndrome d’Usher de type 2

Un patient de 50 ans, marocain, sans profession, aux antécédents d'appendicectomie, il était le fils unique d'un mariage consanguin. Il était affecté d'une surdité congénitale modérée lui permettant l'acquisition du langage. Il a développé secondairement une baisse de l'acuité visuelle de l'œil droit vers l'âge de 16 ans puis de l'œil gauche deux ans plus tard d'aggravation progressive. Ni ses parents et ni ses trois fils ne présentaient de telles anomalies cliniques. L'examen ophtalmologique trouvait une acuité visuelle limité à mouvement des doigts au niveau de l'œil droit et à compte les doits à 1 mètre au niveau de l'œil gauche, l'examen à la lampe à fente trouvait une cataracte sous capsulaire postérieure plus obturante à droite qu'à gauche avec au fond de l'œil un rétrécissement artériolaire diffus, une papille pâle et surtout, la présence de migrations pigmentées intrarétiniennes sombres réparties sur tout le fond d'œil (Figure 1). L'examen ORL était normal tandis que, l'audiométrie tonale montrait une perte audiométrique moyenne du second degré entre 50 et 70 décibels. L'examen neurologique était normal, ne montrant notamment pas de trouble cognitif, de syndrome vestibulaire, cérébelleux ou d'ataxie proprioceptive. L'électrorétinogramme (ERG) montrait une extinction globale en ambiance photopique aussi bien que scotopique, confirmant la dégénérescence rétinienne globale avancée. Le patient a bénéficié d'une chirurgie de cataracte par phacoémulsifition avec mise en place d'un implant oculaire au niveau des deux yeux, ce qui a permis d'améliorer son acuité visuelle à 1/10 au niveau de l'œil droit et à 2/10 au niveau de l'œil gauche.

## Discussion

L'association d'une rétinopathie pigmentaire et d'une surdité congénitale de perception dans un contexte de consanguinité fait suggérer chez notre patient le diagnostic de syndrome d'Usher. [1]. La présence d'une surdité congénitale modérée avec acquisition du langage, des réponses vestibulaires normales et une rétinopathie pigmentaire qui débute vers l'âge adulte jeune sont en faveur du type 2 ce syndrome. Sachant que le type 1 se caractérise par la profondeur et le caractère congénital de la surdité de perception empêchant l'acquisition de la parole. Les troubles vestibulaires entraînent souvent une ataxie discrète et retardent l'acquisition de la marche. La rétinopathie pigmentaire débute dans l'enfance, souvent dès les dix premières années [2]. Notre observation se distingue du type 3 où la surdité est progressive avec une fonction vestibulaire généralement altérée.

Le tableau clinique s'explique par une dégénérescence progressive des cellules photoréceptrices en bâtonnets et au niveau cochléaire, à une perte étendue des cellules ciliaires de l'organe de Corti et des cellules ganglionnaires spiralées avec atrophie des vascularis stria. Les troubles de l'équilibre et/ ou de la coordination sont inconstants dans le type1, alors qu'un retard mental ou des manifestations psychiatriques peuvent être présents dans les types 2 et 3 [3,4]. L'étude génétique du syndrome d'Usher conclut à l'hétérogénéité de la maladie, les trois types cliniques pouvant se diviser en plusieurs sous-types génétiques [5,6]. Les différentes mutations géniques sont connues pour provoquer les différentes formes; à ce jour, toutes les mutations ne sont pas connues. Les recherches récentes portent sur l'identification de nouveaux gènes, sur la fonction des protéines correspondantes et sur les interactions entre les protéines. Huit gènes et deux loci sont actuellement connus, correspondant à la totalité des cas décrits [7]. Les deux gènes majeurs sont MYO7A et USH2A [8]. Les études ont montré que toutes les protéines actuellement connues du syndrome d'Usher type 1 agissent sur la cohésion des stéréocils qui se trouvent dans les cellules auditives de l'oreille interne et dans les différentes structures des photorécepteurs. Toutes les formes du syndrome d'Usher sont transmises par transmission autosomique récessive [9]. Les deux parents sont porteurs sains; le risque est alors de 25 % à chaque grossesse. Les couples ayant déjà un enfant atteint peuvent bénéficier d'un diagnostic prénatal, au sein de la même famille les symptômes peuvent être de gravité variable. La possibilité d'une consanguinité a été décrite.

Le diagnostic moléculaire est aujourd'hui possible, guidé par la clinique, mais coûteux à réaliser [7]. Les données neuroradiologiques récentes ont permis de décrire des lésions inconstantes telles qu'une atrophie cérébelleuse ou plus rarement du tronc cérébral ou du cortex occipital [10]. L'électrorétinogramme (ERG) est un examen complémentaire très utile pour poser le diagnostic de rétinopathie pigmentaire débutante, mais est de peu d'intérêt diagnostique dans les cas évolués. Sur le plan ophtalmologique, les capacités visuelles restantes doivent être optimisées et une consultation orthoptique et de basse vision organisée [11]. Les complications sont dominées par la cataracte et l'œdème maculaire cystoïde. Pour la cataracte, une étude a montré le bénéfice de l'intervention pour des opacités parfois modérées et a prouvé l'augmentation de l'acuité visuelle en fonction de l'état de l'atteinte maculaire, comme ce fut le cas chez notre patient. L'incidence de l'opacification secondaire de la membrane capsulaire est assez fréquente, mais la fréquence des complications postopératoires œdémateuses est faible [12]. En cas de baisse de l'acuité visuelle due à un œdème maculaire cystoïde, complication classique de cette affection, la prise d'acétazolamide peut parfois améliorer la vision pendant un certain temps [13].

## Conclusion

Les rétinopathies pigmentaires constituent un groupe d'affections conduisant à un handicap visuel souvent sévère. L'association à une surdité congénitale définit le syndrome d'Usher. La complexité des mécanismes physiopathologiques et la grande hétérogénéité génétique constituent une difficulté pour une approche thérapeutique globale.

## Conflits d’intérêts

Les auteurs ne déclarent aucun conflit d'intérêts.

## References

[1] Fishman GA, Kumar A, Joseph ME, Torok N, Aderson RJ (1983). Usher's syndrome: Ophthalmic and neuro-otologic findings suggesting genetic heterogeneity. Arch Ophthalmol..

[2] Keats BJ, Corey DP (1999). The Usher syndromes. Am J Med Genet..

[3] Tamayo ML, Maldonado C, Plaza SL, Alvira GM, Tamayo GE, Zambrano M (1996). Neuroradiology and clinical aspects of Usher syndrome. Clin Genet..

[4] Viala A, Nicot T, Levy F, Vacheron MN (2009). A case of Usher's syndrome associated with psychotic symptoms: diagnosis and follow-up in a psychiatric unit. Ecephale..

[5] Keats BJ, Savas S (2004). Genetic heterogeneity in Usher syndrome. Am J Med Genet A..

[6] Kimberling WJ, Moller CG, Davenport S, Priluck IA, Beighton PH, Greenberg J (1992). Linkage of Usher syndrome type I gene (USH1B) to the long arm of chromosome 11. Genomics..

[7] Blanchet C, Roux AF, Hamel C, Ben Salah S, Artières F (2007). Usher type I syndrome in children: genotype/phenotype correlation and cochlear implant benefits. Rev Laryngol Otol Rhinol (Bord)..

[8] Maubaret C, Griffoin JM, Arnaud B, Hamel C (2005). Novel mutations in MYO7A and USH2A genes in Usher Syndrome. Ophtalmic Genet..

[9] Bonneau D, Raymond F, Kremer C, Klossek JM, Kaplan J, Patte F (1993). Usher syndrome type I associated with bronchiectasis and immotile nasal cilia in two brothers. J Med Genet..

[10] Drouet A, Swalduz B, Guilloton L, Faivre A, Felten D (2003). Usher syndrome: a case report. Rev Neurol (Paris)..

[11] Paskowitz DM, Lavail MM, Duncan JL (2006). Light and inherited retinal degeneration. Br J Ophthalmol..

[12] Jackson H, Garway-Heath D, Rosen P, Bird AC, Tuft SJ (2001). Outcome of cataract surgery in patients with retinitis pigmentosa. Br J Ophthalmol..

[13] Cox SN, Hay E, Bird AC (1988). Treatment of chronic macular edema with acetazolamide. Arch Ophthalmol..

